# Association of Normal-Weight Central Obesity With All-Cause and Cause-Specific Mortality Among Postmenopausal Women

**DOI:** 10.1001/jamanetworkopen.2019.7337

**Published:** 2019-07-24

**Authors:** Yangbo Sun, Buyun Liu, Linda G. Snetselaar, Robert B. Wallace, Bette J. Caan, Thomas E. Rohan, Marian L. Neuhouser, Aladdin H. Shadyab, Rowan T. Chlebowski, JoAnn E. Manson, Wei Bao

**Affiliations:** 1 Department of Epidemiology, College of Public Health, University of Iowa, Iowa City; 2 Division of Research, Kaiser Permanente Northern California, Oakland; 3 Department of Epidemiology and Population Health, Albert Einstein College of Medicine, Bronx, New York; 4 Cancer Prevention Program, Fred Hutchinson Cancer Research Center, Seattle, Washington; 5 Department of Family Medicine and Public Health, School of Medicine, University of California, San Diego, La Jolla; 6 Department of Medical Oncology and Therapeutics Research, City of Hope National Medical Center Duarte, Duarte, California; 7 Division of Preventive Medicine, Department of Medicine, Brigham and Women’s Hospital, Harvard Medical School, Boston, Massachusetts; 8 Obesity Research and Education Initiative, University of Iowa, Iowa City; 9 Fraternal Order of Eagles Diabetes Research Center, University of Iowa, Iowa City

## Abstract

**Importance:**

Current public health guidelines for obesity prevention and control focus on promoting a normal body mass index (BMI), rarely addressing central obesity, which is reflected by high waist circumference (WC) and common in the general population. Studies of the association of normal-weight central obesity with long-term health outcomes are sparse.

**Objective:**

To examine associations of normal-weight central obesity with all-cause and cause-specific mortality in postmenopausal women in the United States.

**Design, Setting, and Participants:**

A nationwide prospective cohort study of 156 624 postmenopausal women enrolled in the Women’s Health Initiative at 40 clinical centers in the United States between 1993 and 1998. These women were observed through February 2017. Data analysis was performed from September 15, 2017, to March 13, 2019.

**Exposures:**

Different combinations of BMI (calculated as weight in kilograms divided by height in meters squared; normal weight: BMI, 18.5-24.9; overweight: BMI, 25.0-29.9; and obesity: BMI, ≥30) and WC (normal: WC, ≤88 cm and high: WC, >88 cm).

**Main Outcomes and Measures:**

Mortality from all causes, cardiovascular disease, and cancer.

**Results:**

Of the 156 624 women (mean [SD] age, 63.2 [7.2] years), during 2 811 187 person-years of follow-up, 43 838 deaths occurred, including 12 965 deaths from cardiovascular disease (29.6%) and 11 828 deaths from cancer (27.0%). Compared with women with normal weight and no central obesity and adjusted for demographic characteristics, socioeconomic status, lifestyle factors, and hormone use, the hazard ratio for all-cause mortality was 1.31 (95% CI, 1.20-1.42) among women with normal weight and central obesity, 0.91 (95% CI, 0.89-0.94) among women with overweight and no central obesity, 1.16 (95% CI, 1.13-1.20) for women with overweight and central obesity, 0.93 (95% CI, 0.89-0.94) for women with obesity and no central obesity, and 1.30 (95% CI, 1.27-1.34) for women with obesity and central obesity. Compared with normal weight without central obesity, normal-weight central obesity was associated with higher risk of cardiovascular disease mortality (hazard ratio, 1.25; 95% CI, 1.05-1.46) and cancer mortality (hazard ratio, 1.20; 95% CI, 1.01-1.43).

**Conclusions and Relevance:**

Normal-weight central obesity in women was associated with excess risk of mortality, similar to that of women with BMI-defined obesity with central obesity. These findings underscore the need for future public health guidelines to include the prevention and control of central obesity, even in individuals with normal BMI.

## Key Points

**Question:**

How is normal-weight central obesity associated with risk of mortality compared with other anthropometric phenotypes?

**Findings:**

In this cohort study of 156 624 postmenopausal US women enrolled in the Women’s Health Initiative study, normal-weight central obesity was associated with higher risk of all-cause, cardiovascular disease, and cancer mortality compared with normal weight without central obesity. The magnitude of this association was similar to that of obesity with central obesity and higher than that of other anthropometric phenotypes defined by body mass index and waist circumference.

**Meaning:**

Normal-weight central obesity is an underrecognized, high-risk phenotype for mortality.

## Teaser

This cohort study uses data from women enrolled in the Women’s Health Initiative (WHI) to examine the associations of normal-weight central obesity with all-cause and cause-specific mortality among postmenopausal women in the United States.

## Introduction

Although body mass index (BMI, calculated as weight in kilograms divided by height in meters squared) is the standard measure used to define obesity in clinical and public health guidelines,^1,2,3^ an inherent limitation is that BMI does not distinguish body shape or body fat distribution.^4^ Central obesity, characterized by relatively high abdominal fat distribution, has been associated with higher risk of mortality, independent of BMI.^5^ Even among individuals with normal weight (BMI, <25.0), those with central obesity may be at increased risk of mortality because of excessive abdominal fat accumulation.^6^ However, individuals who have normal BMI with central obesity are usually neglected in clinical guidelines. In the most recent obesity management guidelines by the American Heart Association, the American College of Cardiology, and the Obesity Society,^1^ measuring central obesity was recommended among people who have overweight or class I obesity (BMI, 25.0-34.9) but not among people of normal weight owing to lack of available evidence regarding risk evaluation in this group. Moreover, individuals with normal-weight central obesity receive little attention in the setting of risk reduction strategies, such as lifestyle modifications and other interventions. Because central obesity is common among US adults, including those with normal BMI,^7^ it is important to evaluate long-term health risks among people with normal weight and central obesity.

Our hypothesis was that women with normal weight (defined by BMI) and central obesity (defined by waist circumference [WC]) and women with other BMI/WC combinations were at higher risk of mortality compared with women with normal weight and no central obesity. We examined associations of different combinations of BMI and central obesity with all-cause and cause-specific mortality and assessed the magnitude of risk from normal weight with central obesity by comparing it with the risk from other combinations of BMI and central obesity in a large, prospective cohort of postmenopausal women in the United States.

## Methods

### Study Population

The Women’s Health Initiative (WHI) study design has been previously described in detail.^8^ Briefly, between 1993 and 1998, postmenopausal women aged 50 to 79 years at study entry were recruited through 40 clinical centers into either a randomized clinical trial (RCT) component (n = 68 132) or an observational study (OS) component (n = 93 676 women). The RCTs consisted of 4 trials including 1 dietary modification trial, 2 hormonal therapy trials, and 1 calcium and vitamin D trial. The RCTs and OS were closed between 2004 and 2005, and participants were invited to continue being observed in the WHI Extension Study, which currently has follow-up data through February 2017. In the present study, we included participants in the RCT and OS components. Written informed consent was obtained from each participant. Institutional review board approval was obtained from all participating institutions. The present study was performed and reported in accordance with the Strengthening the Reporting of Observational Studies in Epidemiology (STROBE) reporting guideline for cohort studies.

### Anthropometric Measures

Height, weight, and WC were measured in the study clinic at baseline.^8^ Weight was measured to the nearest 0.1 kg on a balance beam scale with the participant dressed in indoor clothing without shoes. Height was measured to the nearest 0.1 cm using a wall-mounted stadiometer. Trained staff at each WHI clinic used tape measures to measure WC at the natural waist or narrowest part of the torso to the nearest 0.5 cm.

Body mass index was classified as follows: (1) normal weight, BMI from 18.5 to 24.9; (2) overweight, BMI from 25.0 to 29.9; and (3) obesity, BMI 30.0 or more.^9^ Central obesity was defined as WC higher than 88 cm.^10^ Obesity patterns were categorized into 6 groups on the basis of combinations of BMI and WC categories as follows: (1) normal weight without central obesity (BMI, 18.5-24.9; WC, ≤88 cm), (2) normal weight with central obesity (BMI, 18.5-24.9; WC, >88 cm), (3) overweight without central obesity (BMI, 25.0-29.9; WC, ≤88 cm), (4) overweight with central obesity (BMI, 25.0-29.9; WC, >88 cm), (5) obese without central obesity (BMI, ≥30.0; WC, ≤88 cm), and (6) obese with central obesity (BMI, ≥30.0; WC, >88 cm).

### Ascertainment of Death

Mortality end points for this study included all-cause mortality (primary outcome), cardiovascular disease (CVD) mortality, and cancer mortality. Death from CVD included death from all diseases of the circulatory system (*International Statistical Classification of Diseases and Related Health Problems, Tenth Revision *codes I00-I99). Death from cancer included death from all cancer (codes C01 to C99). Deaths were ascertained by review of medical records and death certificates at the WHI Clinical Coordinating Center and by linkage to the National Death Index.^11^ All adjudicators were masked to RCT randomization assignment. Ascertainment of outcomes was complete as of February 28, 2017.

### Other Covariate Assessments

Information on demographic characteristics (ie, age, race/ethnicity), individual socioeconomic status (ie, education level, annual income), and lifestyle (ie, smoking status, physical activity, alcohol intake, total energy intake, overall diet quality, and past hormone use [ie, unopposed estrogen use and estrogen plus progestin use]) was collected at baseline through self-reports. Overall diet quality was indicated by the Alternative Healthy Eating Index 2010,^12^ scored on the basis of the intake of 11 components including vegetables, fruit, whole grains, sugar-sweetened beverages and fruit juices, nuts and legumes, red/processed meat, trans fat, long-chain (omega-3) fats (ie, eicosapentaenoic acid and docosahexaenoic acid), polyunsaturated fatty acids, sodium consumption, and alcohol consumption. Recreational moderate to vigorous intensity physical activity, including walking, was assessed by questionnaire, and metabolic equivalent of task hours per week of recreational physical activity were calculated for each participant.^8,13^ Neighborhood socioeconomic status (NSES) was assessed by using data from the 2000 US Census at the census tract level, as described in previous publications.^14^ The NSES index was scaled to range from 0 to 100 for census tracts; higher scores indicated more affluent tracts. When categorizing each categorical covariate, we created a category for missing data where needed.

### Statistical Analysis

Comparisons of covariates among different groups of women with different combinations of BMI and WC were performed using analysis of variance for continuous variables and χ^2^ test for categorical variables. Cox proportional hazards regression models were used to estimate hazard ratios (HRs) and 95% CIs for risk of all-cause mortality associated with different BMI/WC patterns and cause-specific hazard models for risk of cause-specific mortality. Person-years were calculated from the date of the anthropometric measurement at baseline until death, the last National Death Index search date, or the follow-up through February 28, 2017, whichever came first. Multivariable models were constructed in several stages. Model 1 was adjusted for age at baseline and race/ethnicity. Model 2 was additionally adjusted for education level, annual income, WHI component (ie, RCT or OS), NSES (in quartiles), unopposed estrogen use, and estrogen plus progestin use. Model 3 was additionally adjusted for smoking status, physical activity, alcohol intake, total energy intake (in quartiles), and score on the Alternative Healthy Eating Index 2010 (in quartiles).

We evaluated if the associations varied by age (<65 vs ≥65 years), race/ethnicity (white vs black, Hispanic, American Indian/Alaskan Native, Asian/Pacific Islander, or other), education (<college vs ≥college), annual income (<$50 000 vs ≥$50 000), smoking status (never smoked vs ever smoked), physical activity (metabolic equivalent of task hours per week, <10 vs ≥10), diet quality (Alternative Healthy Eating Index 2010 score, ≤50 vs >50), NSES (≤75 vs >75), unopposed estrogen use (never used vs ever used), and estrogen plus progestin use (never used vs ever used). We first conducted interaction tests via multiplicative interaction terms in the multivariable models, and when significant interactions were detected, we showed data in different strata. In sensitivity analyses, we repeated the analyses by (1) excluding women from RCTs; (2) restricting the analysis to white women; and (3) excluding women with major comorbidities at baseline (eTables 1-3 in the Supplement). We performed additional analysis by using waist-to-hip ratio of 0.85 or higher to define central obesity.^15^

All statistical analyses were conducted using SAS version 9.4 (SAS Institute). All statistical tests were based on prespecified hypotheses, and therefore, there was no adjustment for multiple testing. All tests were 2-sided with statistical significance set at *P* < .05.

## Results

Of participants in the WHI RCT (n = 68 132) and OS (n = 93 676) components, 159 792 women (RCT, 67 584 [99.2%]; OS, 92 208 [98.4%]) had information on BMI and WC. We excluded 1393 women who had BMI lower than 18.5 and 1775 women who died within 3 years of the baseline visit, leaving 156 624 women in the final study cohort (RCT, 66 674 [42.6%]; OS, 89 950 [57.4%]).

Of the 156 624 women (mean [SD] age, 63.2 [7.2] years) included in this study, 1390 had normal-weight central obesity, accounting for 0.9% among all women and 2.6% among women with normal weight. During the 2 811 187 person-years of follow up, 43 838 deaths occurred, including 12 965 deaths from CVD (29.6%), 11 828 deaths from cancer (27.0%), and 19 045 deaths from other causes (43.4%). Across BMI categories, women with central obesity were more likely than women without central obesity to be older and nonwhite, with less education, lower income, and lower NSES compared with women without central obesity (Table 1). Women with central obesity were more likely than women without central obesity to be participants from the WHI RCTs, noncurrent users of hormones, and current smokers and to have lower physical activity levels, higher total energy intake, and lower diet quality.

**Table 1.** Baseline Characteristics of the Study Population (N = 156 624) According to Different Combinations of Body Mass Index and WC^a^

Variable	No. (%)								
	Normal Weight			Overweight			Obesity		
	Normal WC	High WC	*P* Value	Normal WC	High WC	*P* Value	Normal WC	High WC	*P *Value
Participants	52 735 (33.7)	1390 (0.9)	NA	36 337 (23.2)	18 572 (11.9)	NA	4957 (3.2)	42 633 (27.2)	NA
Age at baseline, mean (SD), y	63.3 (7.4)	65.4 (7.1)	<.001	63.1 (7.2)	64.5 (7.1)	<.001	62.2 (7.2)	62.6 (6.9)	<.001
Race/ethnicity									
White	46 031 (87.3)	1223 (88.0)	<.001	29 833 (82.1)	15 754 (84.8)	<.001	3544 (71.5)	32 914(77.2)	<.001
Black	2124 (4.0)	83 (6.0)		3084 (8.5)	1555 (8.4)		844 (17.0)	6473(15.2)	
Hispanic	1523 (2.9)	26 (1.9)		1790 (4.9)	629 (3.4)		388 (7.8)	1963(4.6)	
Other	2911 (5.5)	52 (3.7)		1541 (4.2)	584 (3.1)		174 (3.5)	1185 (2.8)	
Missing	146 (0.3)	6 (0.4)		89 (0.2)	50 (0.3)		7 (0.1)	98 (0.2)	
Education									
≤High school	13 959 (26.5)	461 (33.2)	<.001	11 521 (31.7)	6480 (34.9)	<.001	1756 (35.4)	16 876 (39.6)	<.001
Some college	13 641 (25.9)	365 (26.3)		10 061 (27.7)	5256 (28.3)		1361 (27.5)	12 446 (29.2)	
College	6962 (13.2)	145 (10.4)		3970 (10.9)	1909 (10.3)		465 (9.4)	3465 (8.1)	
Postgraduate	17 789 (33.7)	413 (29.7)		10 496 (28.9)	4787 (25.8)		1331 (26.9)	9526 (22.3)	
Missing	384 (0.7)	6 (0.4)		289 (0.8)	140 (0.8)		44 (0.9)	320 (0.8)	
Income, $									
<20 000	5991 (11.4)	268 (19.3)	<.001	4900 (13.5)	3385 (18.2)	<.001	792 (16.0)	9034 (21.2)	<.001
20 000-49 999	20 190 (38.3)	605 (43.5)		15 183 (41.8)	8285 (44.6)		2069 (41.7)	19 146 (44.9)	
≥50 000	22 774 (43.2)	417 (30.0)		13 838 (38.1)	5723 (30.8)		1742 (35.1)	11 786 (27.7)	
Missing	3780 (7.2)	100 (7.2)		2416 (6.7)	1179 (6.4)		354 (7.1)	2667 (6.3)	
NSES, mean (SD)	77.3 (7.7)	75.9 (8.1)	<.001	75.9 (8.3)	75.4 (8.7)	<.001	74.0 (9.7)	73.7 (9.5)	.02
WHI component									
Randomized clinical trials	17 493 (33.2)	532 (38.3)	<.001	15 399 (42.4)	8535 (46.0)	<.001	2324 (46.9)	22 391 (52.5)	<.001
Observational study	35 242 (66.8)	858 (61.7)		20 938 (57.6)	10 037 (54.0)		2633 (53.1)	20 242 (47.5)	
Unopposed estrogen usage status									
Never used	33 770 (64.0)	862 (62.0)	<.001	22 946 (63.2)	11 727 (63.1)	<.001	3166 (63.9)	28 366 (66.5)	<.001
Past user	6478 (12.3)	228 (16.4)		4502 (12.4)	2683 (14.5)		560 (11.3)	5692 (13.4)	
Current user	12 487 (23.7)	300 (21.6)		8889 (24.5)	4162 (22.4)		1231 (24.8)	8575 (20.1)	
Estrogen plus progestin usage status									
Never used	35 285 (66.9)	1063 (76.5)	<.001	26 294 (72.4)	14 526 (78.2)	<.001	3810 (79.9)	34 778 (81.6)	<.001
Past user	5020 (9.5)	106 (7.6)		3288 (9.1)	1566 (8.4)		386 (7.8)	3029 (7.1)	
Current user	12430 (23.6)	221 (15.9)		6755 (18.6)	2480 (13.4)		761 (15.4)	4826 (11.3)	
Smoking status									
Never smoked	26 958 (51.1)	591 (42.5)	<.001	18 908 (52.0)	8635 (46.5)	<.001	2812 (56.7)	21 143 (49.6)	<.001
Past smoker	21 110 (40.0)	615 (44.2)		14 854 (40.9)	8091 (43.6)		1857 (37.5)	18 464 (43.3)	
Current smoker	3995 (7.6)	166 (11.9)		2104 (5.8)	1601 (8.6)		226 (4.6)	2454 (5.8)	
Missing	672 (1.3)	18 (1.3)		471 (1.3)	245 (1.3)		62 (1.3)	572 (1.3)	
Physical activity, MET-h/wk									
<10	21 218 (40.2)	737 (53.0)	<.001	17 375 (47.8)	10 438 (56.2)	<.001	2743 (55.3)	28 415 (66.7)	<.001
≥10	29 430 (55.8)	591 (42.5)		17 108 (47.1)	7238 (39.0)		1967 (39.7)	12 044 (28.3)	
Missing	2087 (4.0)	62 (4.5)		1854 (5.1)	896 (4.8)		247 (5.0)	2174 (5.1)	
Total energy intake, mean (SD), kcal/d	1539 (559)	1625 (610)	<.001	1607 (609)	1662 (644)	<.001	1664 (664)	1796 (737)	<.001
Alcohol intake									
Nondrinker	16 422 (31.1)	509 (36.6)	<.001	13 151 (36.2)	7390 (39.8)	<.001	2230 (45.0)	21 917 (51.4)	<.001
Moderate drinking	26 157 (49.6)	596 (42.9)		17 929 (49.3)	8260 (44.5)		2226 (44.9)	16 494 (38.7)	
Heavy drinking	8625 (16.4)	243 (17.5)		4191 (11.5)	2368 (12.8)		337 (6.8)	2894 (6.8)	
Missing	1531 (2.9)	42 (3.0)		1066 (2.9)	554 (3.0)		164 (3.3)	1328 (3.1)	
AHEI-2010 score, mean (SD)	52.4 (11.2)	49.9 (11.3)	<.001	50.2 (10.8)	48.6 (10.7)	<.001	48.82 (10.5)	46.6 (10.4)	<.001

Abbreviations: AHEI, Alternative Healthy Eating Index; MET-h, metabolic equivalent of task hours; NA, not available; NSES, neighborhood socioeconomic status; WC, waist circumference; WHI, Women’s Health Initiative.

^a^ Body mass index calculated as weight in kilograms divided by height in meters squared and categorized as normal weight (18.5-24.9), overweight (25.0-29.9), and obesity (≥30.0). Waist circumference categorized as normal (≤88 cm) and high (>88 cm).

After adjustment for demographic characteristics, NSES, lifestyle factors, and hormone use, women with central obesity in each BMI category were at increased risk of all-cause mortality compared with women with normal weight and no central obesity, while women with overweight or obesity and no central obesity were at a slightly lower risk of all-cause mortality (Table 2). The HRs for all-cause mortality were 1.31 (95% CI, 1.20-1.42; *P* < .001) among women with normal weight and central obesity, 1.16 (95% CI, 1.13-1.20; *P* < .001) among women with overweight and central obesity, and 1.30 (95% CI, 1.27-1.34; *P* < .001) among women with obesity and central obesity. It is notable that women with normal-weight central obesity had a similar risk as women with obesity and central obesity, and the risk was 13% to 44% higher than among women with any other BMI/WC combination, including women with overweight and no central obesity (HR, 0.91; 95% CI, 0.89-0.94; *P* < .001) and women with obesity and no central obesity (HR, 0.93; 95% CI, 0.87-0.99; *P* = .01). Results of defining central obesity as waist-hip ratio of 0.85 or larger were largely similar. The corresponding HRs were 1.33 (95% CI, 1.27-1.39) for normal weight women with central obesity compared with normal weight women without central obesity (eTable 4 in the Supplement). Patterns for CVD, cancer, and non-CVD/noncancer mortality were similar. Women with normal weight and central obesity had a higher risk of cardiovascular disease (HR, 1.24; 95% CI, 1.05-1.46) and cancer mortality (HR, 1.20; 95% CI, 1.01-1.43) compared with women with normal weight and no central obesity.

**Table 2.** Association of Different Combinations of Body Mass Index and WC Status With All-Cause and Cause-Specific Mortality Among 156 624 Postmenopausal Women^a^

Model^b^	HR (95% CI)					
	Normal Weight		Overweight		Obesity	
	Normal WC	High WC	Normal WC	High WC	Normal WC	High WC
Participants, No. (%)	52 735 (33.7)	1390 (0.9)	36 337 (23.2)	18 572 (11.9)	4957 (3.2)	42 633 (27.2)
**Any Cause Mortality**						
No.	13 718	562	8722	6348	1093	13 395
Model 1	1 [Reference]	1.43 (1.31-1.55)	0.92 (0.90-0.95)	1.25 (1.21-1.28)	0.93 (0.87-0.99)	1.40 (1.36-1.43)
Model 2	1 [Reference]	1.39 (1.28-1.51)	0.91 (0.88-0.93)	1.20 (1.17-1.24)	0.90 (0.85-0.96)	1.32 (1.29-1.35)
Model 3	1 [Reference]	1.31 (1.20-1.42)	0.91 (0.89-0.94)	1.16 (1.13-1.20)	0.93 (0.87-0.99)	1.30 (1.27-1.34)
**CVD Mortality**						
No. (%)	3671 (26.8)	149 (26.5)	2534 (29.1)	1954 (30.8)	338 (30.9)	4319 (32.2)
Model 1	1 [Reference]	1.35 (1.15-1.60)	1.00 (0.95-1.05)	1.39 (1.32-1.47)	1.08 (0.96-1.20)	1.70 (1.63-1.78)
Model 2	1 [Reference]	1.30 (1.10-1.53)	0.97 (0.92-1.02)	1.32 (1.25-1.39)	1.02 (0.91-1.14)	1.55 (1.48-1.63)
Model 3	1 [Reference]	1.24 (1.05-1.46)	0.97 (0.93-1.03)	1.28 (1.21-1.35)	1.04 (0.93-1.16)	1.53 (1.46-1.61)
**Cancer Mortality**						
No. (%)	3708 (27.0)	133 (23.7)	2441 (28.0)	1675 (26.4)	303 (27.7)	3568 (26.6)
Model 1	1 [Reference]	1.33 (1.12-1.58)	0.95 (0.90-1.01)	1.25 (1.18-1.33)	0.92 (0.82-1.03)	1.29 (1.23-1.35)
Model 2	1 [Reference]	1.30 (1.09-1.54)	0.94 (0.90-0.99)	1.22 (1.15-1.30)	0.90 (0.80-1.02)	1.24 (1.18-1.30)
Model 3	1 [Reference]	1.20 (1.01-1.43)	0.96 (0.92-1.02)	1.19 (1.12-1.26)	0.96 (0.85-1.08)	1.26 (1.20-1.33)
**Other Cause Mortality**						
No. (%)	6339 (46.2)	280 (48.9)	3747 (43.0)	2719 (42.8)	452 (41.4)	5508 (41.1)
Model 1	1 [Reference]	1.54 (1.36-1.73)	0.86 (0.83-0.90)	1.16 (1.11-1.22)	0.86 (0.78-0.94)	1.30 (1.25-1.34)
Model 2	1 [Reference]	1.50 (1.33-1.69)	0.85 (0.82-0.89)	1.13 (1.08-1.18)	0.83 (0.76-0.91)	1.24 (1.19-1.28)
Model 3	1 [Reference]	1.42 (1.26-1.60)	0.85 (0.82-0.88)	1.09 (1.04-1.14)	0.84 (0.76-0.93)	1.20 (1.16-1.25)

Abbreviations: CVD, cardiovascular disease; HR, hazard ratio; WC, waist circumference.

^a^ Body mass index calculated as weight in kilograms divided by height in meters squared and categorized as normal weight (18.5-24.9), overweight (25.0-29.9), and obesity (≥30.0). Waist circumference categorized as normal (≤88 cm) and high (>88 cm).

^b^ Model 1 adjusted for age at baseline and race/ethnicity. Model 2 additionally adjusted for education, income, component of Women’s Health Initiative (ie, randomized clinical trial or observational study), neighborhood socioeconomic status, unopposed estrogen usage status, and estrogen plus progesterone usage status. Model 3 additionally adjusted for smoking status, physical activity, total energy intake, and Alternative Healthy Eating Index 2010 score.

Associations of different BMI/WC combinations with all-cause, CVD, and cancer mortality were generally similar across categories of age, race/ethnicity, education, income, NSES, diet quality, smoking status, physical activity, unopposed estrogen use, and estrogen plus progestin use, except that the magnitudes of risk were greater for all-cause mortality among younger women (aged <65 years) (Table 3), for CVD mortality among younger women (aged <65 years) and women with higher diet quality (Table 4), and for cancer mortality among women with lower physical activity levels, women with higher diet quality, and women with lower NSES (Table 5). The results were robust in our sensitivity analyses when we restricted to women from the OS component to avoid changes in risk factor status related to the RCT interventions (eTable 1 in the Supplement), when analyses were limited to white women (eTable 2 in the Supplement), and when women with major comorbidities at baseline were excluded (eTable 3 in the Supplement).

**Table 3.** Stratified Analyses for the Association of Baseline Body Mass Index and WC Status With All-Cause Mortality^a^^,^^b^

Characteristic	HR (95% CI)						*P* Value for Interaction
	Normal Weight		Overweight		Obesity		
	Normal WC	High WC	Normal WC	High WC	Normal WC	High WC	
Age, y							
<65	1 [Reference]	1.84 (1.41-2.39)	0.94 (0.87-1.02)	1.32 (1.20-1.45)	0.92 (0.78-1.09)	1.48 (1.38-1.59)	<.001
≥65	1 [Reference]	1.26 (1.16-1.38)	0.91 (0.89-0.94)	1.15 (1.11-1.19)	0.93 (0.87-0.99)	1.28 (1.25-1.32)	
Race							
White	1 [Reference]	1.30 (1.19-1.43)	0.92 (0.89-0.94)	1.16 (1.12-1.19)	0.92 (0.85-0.98)	1.31 (1.27-1.34)	.10
Nonwhite	1 [Reference]	1.40 (1.08-1.82)	0.93 (0.86-1.01)	1.26 (1.15-1.38)	0.99 (0.87-1.13)	1.36 (1.27-1.46)	
Smoking status							
Never smoked	1 [Reference]	1.39 (1.22-1.59)	0.94 (0.90-0.97)	1.18 (1.13-1.24)	0.95 (0.87-1.04)	1.34 (1.29-1.40)	.39
Ever smoked	1 [Reference]	1.27 (1.13-1.41)	0.86 (0.83-0.89)	1.12 (1.07-1.17)	0.86 (0.78-0.94)	1.18 (1.14-1.22)	
Physical activity, MET-h/wk							
<10	1 [Reference]	1.28 (1.15-1.44)	0.88 (0.85-0.92)	1.13 (1.08-1.18)	0.90 (0.83-0.98)	1.28 (1.24-1.33)	.002
≥10	1 [Reference]	1.26 (1.10-1.45)	0.95 (0.92-0.99)	1.19 (1.14-1.25)	0.97 (0.88-1.07)	1.33 (1.28-1.39)	
Unopposed estrogen use							
Never used	1 [Reference]	1.25 (1.12-1.40)	0.90 (0.87-0.93)	1.15 (1.11-1.20)	0.93 (0.86-1.01)	1.31 (1.27-1.35)	.01
Ever used	1 [Reference]	1.40 (1.23-1.60)	0.93 (0.89-0.98)	1.19 (1.13-1.25)	0.92 (0.83-1.02)	1.30 (1.25-1.36)	
Estrogen plus progesterone use							
Never used	1 [Reference]	1.30 (1.19-1.43)	0.90 (0.88-0.93)	1.16 (1.12-1.20)	0.94 (0.88-1.01)	1.29 (1.26-1.33)	.18
Ever used	1 [Reference]	1.33 (1.08-1.64)	0.95 (0.89-1.01)	1.17 (1.09-1.26)	0.85 (0.72-0.99)	1.36 (1.28-1.44)	
AHEI-2010 score							
≤50	1 [Reference]	1.30 (1.16-1.46)	0.87 (0.83-0.90)	1.11 (1.06-1.15)	0.92 (0.84-0.99)	1.23 (1.19-1.28)	**<**.001
>50	1 [Reference]	1.28 (1.13-1.46)	0.96 (0.92-0.99)	1.23 (1.17-1.29)	0.94 (0.86-1.03)	1.40 (1.35-1.45)	
NSES							
≤75	1 [Reference]	1.43 (1.24-1.64)	0.92 (0.88-0.96)	1.12 (1.07-1.18)	0.89 (0.81-0.98)	1.29 (1.23-1.34)	.23
>75	1 [Reference]	1.28 (1.14-1.44)	0.92 (0.89-0.95)	1.18 (1.13-1.23)	0.93 (0.85-1.02)	1.32 (1.27-1.37)	

Abbreviations: AHEI, Alternative Healthy Eating Index; HR, hazard ratio; MET-h, metabolic equivalent of task hours; NSES, neighborhood socioeconomic status; WC, waist circumference.

^a^ Based on model 3, ie, adjusted for age at baseline, race/ethnicity, education, income, NSES, component of Women’s Health Initiative (ie, randomized clinical trial or observational study), unopposed estrogen usage status, estrogen plus progesterone usage status, smoking status, physical activity, total energy intake, and AHEI-2010 score.

^b^ Body mass index calculated as weight in kilograms divided by height in meters squared and categorized as normal weight (18.5-24.9), overweight (25.0-29.9), and obesity (≥30.0). Waist circumference categorized as normal (≤88 cm) and high (>88 cm).

**Table 4.** Stratified Analyses for the Association of Baseline Body Mass Index and WC Status With Cardiovascular Disease Mortality^a^^,^^b^

Characteristic	HR (95% CI)						*P* Value for Interaction
	Normal Weight		Overweight		Obesity		
	Normal WC	High WC	Normal WC	High WC	Normal WC	High WC	
Age, y							
<65	1 [Reference]	1.54 (0.76-3.11)	1.07 (0.88-1.29)	1.67 (1.35-2.07)	1.03 (0.70-1.50)	2.13 (1.80-2.51)	<.001
≥65	1 [Reference]	1.22 (1.03-1.45)	0.97 (0.92-1.02)	1.25 (1.18-1.33)	1.05 (0.94-1.18)	1.49 (1.42-1.56)	
Race							
White	1 [Reference]	1.27 (1.07-1.51)	0.97 (0.92-1.02)	1.25 (1.18-1.33)	1.05 (0.92-1.19)	1.52 (1.45-1.60)	.04
Nonwhite	1 [Ref]	1.06 (0.61-1.84)	1.08 (0.94-1.24)	1.56 (1.34-1.83)	1.14 (0.90-1.45)	1.79 (1.58-2.03)	
Smoking status							
Never smoked	1 [Reference]	1.11 (0.85-1.46)	1.04 (0.97-1.11)	1.32 (1.22-1.44)	1.10 (0.95-1.28)	1.58 (1.47-1.69)	.19
Ever smoked	1 [Reference]	1.34 (1.09-1.65)	0.87 (0.81-0.94)	1.20 (1.11-1.29)	0.94 (0.79-1.12)	1.39 (1.30-1.49)	
Physical activity, MET-h/wk							
<10	1 [Reference]	1.26 (1.02-1.56)	0.95 (0.88-1.02)	1.26 (1.16-1.36)	1.04 (0.90-1.21)	1.55 (1.46-1.65)	.20
≥10	1 [Reference]	1.19 (0.90-1.56)	1.02 (0.95-1.10)	1.34 (1.22-1.46)	1.04 (0.86-1.24)	1.49 (1.38-1.61)	
Unopposed estrogen use							
Never used	1 [Reference]	1.12 (0.90-1.39)	0.96 (0.90-1.03)	1.24 (1.16-1.33)	1.05 (0.91-1.21)	1.54 (1.45-1.63)	.11
Ever used	1 [Reference]	1.44 (1.13-1.84)	1.00 (0.92-1.08)	1.34 (1.22-1.46)	1.03 (0.85-1.24)	1.53 (1.41-1.65)	
Estrogen plus progesterone use							
Never used	1 [Reference]	1.24 (1.04-1.48)	0.98 (0.93-1.04)	1.28 (1.20-1.36)	1.05 (0.93-1.18)	1.52 (1.44-1.60)	.59
Ever used	1 [Reference]	1.23 (0.79-1.92)	0.95 (0.84-1.07)	1.27 (1.09-1.46)	0.98 (0.72-1.35)	1.61 (1.43-1.82)	
AHEI-2010 score							
≤50	1 [Reference]	1.30 (1.04-1.62)	0.92 (0.86-0.99)	1.19 (1.10-1.28)	1.04 (0.89-1.21)	1.40 (1.32-1.50)	<.001
>50	1 [Reference]	1.12 (0.86-1.45)	1.01 (0.94-1.09)	1.37 (1.26-1.49)	1.04 (0.87-1.23)	1.70 (1.58-1.82)	
NSES							
≤75	1 [Reference]	1.24 (0.95-1.63)	0.96 (0.88-1.04)	1.22 (1.11-1.33)	1.04 (0.88-1.22)	1.47 (1.36-1.59)	.95
>75	1 [Reference]	1.30 (1.04-1.62)	0.99 (0.92-1.06)	1.29 (1.20-1.39)	0.99 (0.84-1.17)	1.57 (1.47-1.67)	

Abbreviations: AHEI, Alternative Healthy Eating Index; HR, hazard ratio; MET-h, metabolic equivalent of task hours; NSES, neighborhood socioeconomic status; WC, waist circumference.

^a^ Based on model 3, ie, adjusted for age at baseline, race/ethnicity, education, income, NSES, component of Women’s Health Initiative (ie, randomized clinical trial or observational study), unopposed estrogen usage status, estrogen plus progesterone usage status, smoking status, physical activity, total energy intake, and AHEI-2010 score.

^b^ Body mass index calculated as weight in kilograms divided by height in meters squared and categorized as normal weight (18.5-24.9), overweight (25.0-29.9), and obesity (≥30.0). Waist circumference categorized as normal (≤88 cm) and high (>88 cm).

**Table 5.** Stratified Analyses for the Association of Baseline Body Mass Index and WC Status With Cancer Mortality^a^^,^^b^

Characteristic	HR (95% CI)						*P* Value for Interaction
	Normal Weight		Overweight		Obesity		
	Normal WC	High WC	Normal WC	High WC	Normal WC	High WC	
Age, y							
<65	1 [Reference]	1.54 (1.02-2.33)	0.94 (0.84-1.06)	1.22 (1.06-1.40)	0.95 (0.75-1.20)	1.22 (1.09-1.36)	.31
≥65	1 [Reference]	1.15 (0.95-1.39)	0.97 (0.92-1.03)	1.18 (1.11-1.26)	0.96 (0.84-1.10)	1.27 (1.20-1.35)	
Race							
White	1 [Reference]	1.17 (0.97-1.40)	0.98 (0.92-1.03)	1.19 (1.12-1.27)	0.90 (0.78-1.03)	1.27 (1.21-1.34)	.27
Nonwhite	1 [Reference]	1.54 (0.96-2.48)	0.89 (0.77-1.03)	1.15 (0.97-1.36)	1.08 (0.86-1.36)	1.19 (1.04-1.36)	
Smoking status							
Never smoked	1 [Reference]	1.44 (1.09-1.92)	0.98 (0.91-1.06)	1.24 (1.13-1.37)	0.99 (0.83-1.18)	1.37 (1.27-1.49)	.17
Ever smoked	1 [Reference]	1.10 (0.88-1.37)	0.91 (0.85-0.98)	1.13 (1.04-1.21)	0.85 (0.72-1.01)	1.09 (1.02-1.16)	
Physical activity, MET-h/wk							
<10	1 [Reference]	1.29 (1.03-1.61)	0.94 (0.87-1.01)	1.14 (1.05-1.23)	0.93 (0.79-1.09)	1.21 (1.14-1.30)	.046
≥10	1 [Reference]	0.98 (0.72-1.32)	0.97 (0.90-1.05)	1.23 (1.13-1.35)	0.98 (0.82-1.18)	1.32 (1.22-1.43)	
Unopposed estrogen use							
Never used	1 [Reference]	1.16 (0.93-1.45)	0.95 (0.89-1.01)	1.18 (1.09-1.27)	0.97 (0.84-1.12)	1.25 (1.17-1.32)	.90
Ever used	1 [Reference]	1.29 (0.98-1.70)	1.00 (0.91-1.08)	1.22 (1.11-1.34)	0.94 (0.76-1.15)	1.32 (1.21-1.43)	
Estrogen plus progesterone use							
Never used	1 [Reference]	1.26 (1.04-1.53)	0.96 (0.90-1.02)	1.21 (1.14-1.30)	0.97 (0.85-1.11)	1.26 (1.19-1.33)	.35
Ever used	1 [Reference]	0.98 (0.64-1.49)	0.98 (0.89-1.09)	1.10 (0.96-1.25)	0.92 (0.71-1.19)	1.30 (1.17-1.44)	
AHEI-2010 score							
≤50	1 [Reference]	1.11 (0.87-1.42)	0.90 (0.83-0.97)	1.13 (1.05-1.23)	0.91 (0.77-1.07)	1.19 (1.11-1.27)	.02
>50	1 [Reference]	1.24 (0.96-1.60)	1.04 (0.96-1.11)	1.26 (1.16-1.38)	1.04 (0.87-1.23)	1.37 (1.27-1.48)	
NSES							
≤75	1 [Reference]	1.54 (1.18-2.01)	1.01 (0.93-1.11)	1.11 (0.99-1.23)	0.97 (0.81-1.17)	1.27 (1.17-1.38)	.02
>75	1 [Reference]	1.02 (0.80-1.32)	0.94 (0.88-1.01)	1.23 (1.14-1.33)	0.91 (0.77-1.08)	1.25 (1.17-1.34)	

Abbreviations: AHEI, Alternative Healthy Eating Index; HR, hazard ratio; MET-h, metabolic equivalent of task hours; NSES, neighborhood socioeconomic status; WC, waist circumference.

^a^ Based on model 3, ie, adjusted for age at baseline, race/ethnicity, education, income, NSES, component of Women’s Health Initiative (ie, randomized clinical trial or observational study), unopposed estrogen usage status, estrogen plus progesterone usage status, smoking status, physical activity, total energy intake, and AHEI-2010 score.

^b^ Body mass index calculated as weight in kilograms divided by height in meters squared and categorized as normal weight (18.5-24.9), overweight (25.0-29.9), and obesity (≥30). Waist circumference categorized as normal (≤88 cm) and high (>88 cm).

## Discussion

In this large prospective cohort study among postmenopausal women with long-term follow-up, women with normal weight and central obesity had an elevated risk of all-cause mortality, similar to the risk among women with obesity and central obesity and higher than the risk among women with any other combination of BMI and WC. Women with normal-weight central obesity had the second highest risk of CVD mortality, with women with obesity and central obesity having the greatest risk.

Although several previous studies have investigated associations of BMI and WC, either separately or in combination, with mortality risk,^16,17,18,19,20^ very few studies have evaluated the risk of mortality among people with normal-weight central obesity or the magnitude of the risk.^6,21^ Our results are generally consistent with those of a study using data from the National Health and Nutrition Examination Survey (NHANES) III that showed that among 7249 women, those with normal-weight central obesity had an elevated risk of all-cause mortality, similar in magnitude to the risk among women with obesity and central obesity.^6^ The risk of CVD mortality among women with normal-weight central obesity compared with women with other obesity patterns in the NHANES study were unclear. Similar results were observed in a UK study among 42 702 participants.^21^ However, neither the NHANES study nor the UK study investigated the association of normal-weight central obesity with cancer mortality. To our knowledge, our study is the largest study with the longest follow-up period investigating the association of normal-weight central obesity with all-cause and CVD mortality and the first study to report the association of normal-weight central obesity with cancer mortality.

There are several explanations for our findings showing that women with normal-weight central obesity were at higher risk of all-cause, CVD, and cancer mortality. First, the adverse effect of visceral fat and the lack of protective effect of muscle mass owing to this combination of BMI and WC may be an explanation. Body mass index is a measure of both fat and fat-free mass, while WC is a measure of abdominal fat accumulation. Individuals with normal weight and a high WC may have excessive visceral fat, and their normal BMI may put them at risk for less muscle mass than counterparts with the same BMI and no central obesity. Previous studies have shown that excessive visceral fat is associated with insulin resistance, hyperinsulinemia, dyslipidemia, and inflammation, which are risk factors for CVD and several types of cancer, including breast cancer and colon cancer.^22,23,24,25,26,27,28^ In contrast, muscle mass is associated with a more favorable metabolic profile, and the lack of muscle mass could lead to the loss of its protective association with adverse health outcomes.^29,30,31^ Second, higher WC with normal BMI could suggest decreased subcutaneous fat on hips and legs for a given amount of visceral fat. The presence of gluteofemoral adipose tissue has been linked to an improved metabolic and cardiovascular risk profile, owing to differential regulation of lower-body fatty acid release and uptake at the level of the adipocyte and resulting in the long-term entrapment of fatty acids in this depot and protection from ectopic fat accumulation.^32^ Thus, a decrease in the protective gluteofemoral adipose tissue among women with normal-weight central obesity could lead to poor overall survival. On the contrary, women with overweight based on BMI may have a greater amount of gluteofemoral adipose tissue associated with an improved metabolic and cardiovascular risk profile, which could partially explain the obesity paradox.^33,34,35,36^

Our findings have important clinical and public health implications. People with normal weight based on BMI, regardless of central obesity status, were generally considered normal in clinical practice according to current guidelines and policy makers. This could lead to a missed opportunity for risk evaluation and intervention programs for a high-risk but neglected subgroup (ie, those with normal-weight central obesity). In the most recent 2013 American Heart Association, American College of Cardiology, and the Obesity Society obesity management guideline,^1^ measuring waist circumference was recommended only in people who have either overweight or class I obesity (BMI, 25.0-34.9) but not among people with normal weight owing to lack of available evidence regarding risk evaluation in this group. This guideline may send the public and clinical professionals a message that people with normal BMI are free of any particular obesity-related risk, while, in fact, they were at an elevated risk of mortality and might need risk reduction programs, such as lifestyle modifications and other interventions.

### Strengths and Limitations

Our study has several strengths, including the large sample size, the prospective study design, which can establish the temporal direction of the associations, and long-term follow-up. Additionally, we had detailed data on confounders that could potentially alter the associations of BMI/WC combinations with risk of mortality; therefore, we could explore the joint associations of BMI and WC comprehensively. Furthermore, the anthropometric measures were taken directly by trained staff instead of through self-reporting. Last, measurements of BMI and WC are widely available and feasible; thus, our results may be applicable to clinical settings worldwide.

We acknowledge that there are several limitations. First, because our participants were older postmenopausal women, our findings may not apply to women at younger age, premenopausal and perimenopausal women, or men. Second, imaging data of adipose tissue was not available for all WHI participants. Therefore, information on body fat distribution was based on anthropometric measures, such as WC, which may be difficult to measure and less accurate in individuals with a BMI of 35 or higher. However, even though it is an indirect measure of abdominal fat, WC has been shown to be associated with risk of death from CVD, cancer, and other causes.^5^ Furthermore, WC has advantages as an easier, more convenient, and less expensive measure, and therefore, measuring WC is much more feasible in practice than expensive body imaging modalities. Third, the cutoff points of BMI from 25.0 to 29.9 to define overweight and BMI of 30.0 and higher to define obesity might not be appropriate for all nonwhite people. However, most participants in our study were white women, and the results were similar in a sensitivity analysis when we restricted to white participants only. Fourth, we only used baseline exposure data, which could not account for the effect of changes in exposure on outcome.

## Conclusions

This large prospective cohort study found that women with normal-weight central obesity were at higher risk of mortality compared with women with normal weight and no central obesity. Women with normal weight and central obesity were at comparable risk as women with BMI-defined obesity and central obesity. Our results highlight the inability of BMI alone to distinguish body shape or body fat distribution, the misclassification of risk because of adiposity that occurs when using BMI as a proxy for fat mass, and the importance of measuring central obesity even among people with normal weight. Furthermore, our results demonstrated that WC can be used in combination with BMI to better stratify patients for mortality risk. Future research is needed to develop and test the effectiveness of interventions to reduce risk owing to excess body fat among people with normal-weight central obesity. Our findings challenge the current paradigm that measurement of abdominal fat is not recommended for individuals with normal BMI.

## Notes

Supplement. **eTable 1. **Baseline BMI and WC Status in Association With All-Cause and Cause-Specific Mortality Among 89 950 Postmenopausal Women in the WHI OS **eTable 2. **Baseline BMI and WC Status in Association With All-Cause and Cause-Specific Mortality Among 129 299 White Women in the WHI **eTable 3. **Baseline BMI and WC Status in Association With All-Cause and Cause-Specific Mortality Among 138 322 Postmenopausal Women Without CVD, Cancer, and Diabetes at Baseline in the WHI **eTable 4. **Baseline BMI and WHR Status in Association With All-Cause and Cause-Specific Mortality Among 156 624 Postmenopausal Women
