# Supplementary material for: Association of Normal-Weight Central Obesity With All-Cause and Cause-Specific Mortality Among Postmenopausal Women
Source: JAMA Netw Open. 2019 Jul 24;2(7):e197337. doi: 10.1001/jamanetworkopen.2019.7337 (PMC6659146; doi:10.1001/jamanetworkopen.2019.7337)
Supplement: Supplement. — eTable 1. Baseline BMI and WC Status in Association With All-Cause and Cause-Specific Mortality Among 89 950 Postmenopausal Women in the WHI OS eTable 2. Baseline BMI and WC Status in Association With All-Cause and Cause-Specific Mortality Among 129 299 White Women in the WHI eTable 3. Baseline BMI and WC Status in Association With All-Cause and Cause-Specific Mortality Among 138 322 Postmenopausal Women Without CVD, Cancer, and Diabetes at Baseline in the WHI eTable 4. Baseline BMI and WHR Status in Association With All-Cause and Cause-Specific Mortality Among 156 624 Postmenopausal Women [file jamanetwopen-2-e197337-s001.pdf]

Supplementary Online Content

Sun Y, Liu B, Snetselaar LG, et al. Association of normal-weight central obesity with all-cause and cause-specific mortality among postmenopausal women. *JAMA Netw Open*. 2019;2(7):e197337. doi:10.1001/jamanetworkopen.2019.7337

eTable 1. Baseline BMI and WC Status in Association With All-Cause and Cause-Specific Mortality Among 89 950 Postmenopausal Women in the WHI OS

eTable 2. Baseline BMI and WC Status in Association With All-Cause and Cause-Specific Mortality Among 129 299 White Women in the WHI

eTable 3. Baseline BMI and WC Status in Association With All-Cause and Cause-Specific Mortality Among 138 322 Postmenopausal Women Without CVD, Cancer, and Diabetes at Baseline in the WHI

eTable 4. Baseline BMI and WHR Status in Association With All-Cause and Cause-Specific Mortality Among 156 624 Postmenopausal Women

This supplementary material has been provided by the authors to give readers additional information about their work.

© 2019 Sun Y et al. *JAMA Network Open.*

eTable 1. Baseline BMI and WC Status in Association With All-Cause and Cause-Specific Mortality Among 89 950 Postmenopausal Women in the WHI OS

	Normal weight (BMI 18.5-24.9 kg/m^2)		Overweight (BMI 25.0-29.9 kg/m^2)		Obesity (BMI ≥30 kg/m^2)	
Causes of death	Normal WC (≤88 cm)	High WC (>88 cm)	Normal WC (≤88 cm)	High WC (>88 cm)	Normal WC (≤88 cm)	High WC (>88 cm)
No. of participants	35,242	858	20,938	10,037	2,633	20,242
Any cause (n)	9,370	361	5,275	3,661	613	6,827
model 1	1.00(ref)	**1.44(1.30, 1.60)**	**0.94(0.91, 0.97)**	**1.29(1.24, 1.34)**	**0.92(0.85, 0.99)**	**1.47(1.43, 1.52)**
model 2	1.00(ref)	**1.40(1.26, 1.55)**	**0.92(0.89, 0.95)**	**1.22(1.17, 1.27)**	**0.88(0.81, 0.96)**	**1.36(1.32, 1.40)**
model 3	1.00(ref)	**1.30(1.17, 1.44)**	**0.92(0.89, 0.95)**	**1.17(1.12, 1.21)**	**0.89(0.82, 0.97)**	**1.32(1.28, 1.37)**
CVD (n)	2,478	91	1,535	1,143	195	2,227
model 1	1.00(ref)	**1.31(1.06, 1.62)**	1.03(0.96, 1.10)	**1.46(1.36, 1.56)**	1.09(0.94, 1.26)	**1.82(1.72, 1.93)**
model 2	1.00(ref)	**1.27(1.03, 1.56)**	0.99(0.93, 1.06)	**1.36(1.27, 1.46)**	1.03(0.89, 1.19)	**1.64(1.54, 1.74)**
model 3	1.00(ref)	1.19(0.97, 1.47)	0.99(0.93, 1.06)	**1.31(1.22, 1.40)**	1.04(0.90, 1.20)	**1.59(1.49, 1.69)**
Cancer (n)	2,514	85	1,435	940	158	1,749
model 1	1.00(ref)	**1.35(1.09, 1.68)**	0.96(0.90, 1.02)	**1.28(1.19, 1.38)**	0.87(0.74, 1.02)	**1.32(1.24, 1.41)**
model 2	1.00(ref)	**1.32(1.06, 1.63)**	0.94(0.88, 1.01)	**1.24(1.15, 1.33)**	**0.85(0.72, 0.99)**	**1.25(1.18, 1.34)**
model 3	1.00(ref)	1.20(0.97, 1.49)	0.95(0.89, 1.02)	**1.18(1.10, 1.28)**	0.88(0.75, 1.04)	**1.24(1.17, 1.33)**
Other causes (n)	4,378	185	2,305	1,578	260	2,851
model 1	1.00(ref)	**1.57(1.35, 1.82)**	**0.88(0.84, 0.93)**	**1.20(1.13, 1.27)**	**0.85(0.75, 0.97)**	**1.38(1.32, 1.45)**
model 2	1.00(ref)	**1.53(1.32, 1.77)**	**0.86(0.82, 0.90)**	**1.14(1.07, 1.20)**	**0.81(0.72, 0.92)**	**1.27(1.21, 1.33)**
model 3	1.00(ref)	**1.42(1.23, 1.65)**	**0.85(0.81, 0.90)**	**1.08(1.02, 1.15)**	**0.81(0.72, 0.92)**	**1.22(1.16, 1.28)**

Model 1: age at baseline and race/ethnicity.
Model 2: model 1+ education, income, NSES, unopposed estrogen usage status, and estrogen + progesterone usage status.
Model 3: model 2 + smoking status, physical activity, total energy intake, and AHEI-2010 score.
Bold values are statistically significant at $p < 0.05$.

© 2019 Sun Y et al. *JAMA Network Open.*

eTable 2. Baseline BMI and WC Status in Association With All-Cause and Cause-Specific Mortality Among 129 299 White Women in the WHI

	Normal weight (BMI 18.5-24.9 kg/m²)		Overweight (BMI 25.0-29.9 kg/m²)		Obesity (BMI ≥30 kg/m²)	
Causes of death	Normal WC (≤88 cm)	High WC (>88 cm)	Normal WC (≤88 cm)	High WC (>88 cm)	Normal WC (≤88 cm)	High WC (>88 cm)
No. of participants	46,031	1223	29,833	15,754	3,544	32,914
Any cause (n)	12,284	500	7,465	5,512	818	10,763
model 1	1.00(ref)	**1.42(1.30, 1.55)**	**0.92(0.90, 0.95)**	**1.23(1.19, 1.27)**	**0.92(0.85, 0.98)**	**1.39(1.35, 1.43)**
model 2	1.00(ref)	**1.38(1.26, 1.51)**	**0.91(0.88, 0.94)**	**1.19(1.16, 1.23)**	**0.89(0.83, 0.95)**	**1.32(1.28, 1.35)**
model 3	1.00(ref)	**1.30(1.19, 1.43)**	**0.92(0.89, 0.94)**	**1.16(1.12, 1.19)**	**0.92(0.85, 0.98)**	**1.31(1.27, 1.34)**
CVD (n)	3,266	135	2,127	1,660	250	3,369
model 1	1.00(ref)	**1.39(1.17, 1.65)**	1.00(0.94, 1.05)	**1.37(1.29, 1.45)**	1.08(0.95, 1.23)	**1.69(1.61, 1.77)**
model 2	1.00(ref)	**1.33(1.12, 1.58)**	0.97(0.92, 1.02)	**1.29(1.22, 1.37)**	1.02(0.90, 1.16)	**1.55(1.47, 1.62)**
model 3	1.00(ref)	**1.27(1.07, 1.51)**	0.97(0.92, 1.02)	**1.25(1.18, 1.33)**	1.05(0.92, 1.19)	**1.52(1.45, 1.60)**
Cancer (n)	3,294	115	2,084	1,457	213	2,874
model 1	1.00(ref)	**1.28(1.07, 1.55)**	0.96(0.91, 1.01)	**1.25(1.18, 1.33)**	**0.86(0.75, 0.99)**	**1.30(1.24, 1.37)**
model 2	1.00(ref)	**1.25(1.04, 1.51)**	0.95(0.90, 1.01)	**1.22(1.15, 1.30)**	**0.84(0.74, 0.97)**	**1.25(1.19, 1.31)**
model 3	1.00(ref)	1.17(0.97, 1.40)	0.98(0.92, 1.03)	**1.19(1.12, 1.27)**	0.90(0.78, 1.03)	**1.27(1.21, 1.34)**
Other causes (n)	5,724	250	3,254	2,395	355	4,520
model 1	1.00(ref)	**1.52(1.34, 1.73)**	**0.86(0.83, 0.90)**	**1.15(1.10, 1.21)**	**0.86(0.77, 0.96)**	**1.29(1.24, 1.34)**
model 2	1.00(ref)	**1.49(1.31, 1.69)**	**0.85(0.82, 0.89)**	**1.13(1.07, 1.18)**	**0.84(0.75, 0.93)**	**1.24(1.19, 1.29)**
model 3	1.00(ref)	**1.41(1.24, 1.60)**	**0.85(0.82, 0.89)**	**1.09(1.03, 1.14)**	**0.85(0.77, 0.95)**	**1.21(1.16, 1.26)**

Model 1: age at baseline.
Model 2: model 1+ education, income, NSES, unopposed estrogen usage status, and estrogen + progesterone usage status.
Model 3: model 2 + smoking status, physical activity, total energy intake, and AHEI-2010 score.
Bold values are statistically significant at $p < 0.05$.

© 2019 Sun Y et al. *JAMA Network Open.*

eTable 3. Baseline BMI and WC Status in Association With All-Cause and Cause-Specific Mortality Among 138 322 Postmenopausal Women Without CVD, Cancer, and Diabetes at Baseline in the WHI

	Normal weight (BMI 18.5-24.9 kg/m^2)		Overweight (BMI 25.0-29.9 kg/m^2)		Obesity (BMI ≥30 kg/m^2)	
Causes of death	Normal WC (≤88 cm)	High WC (>88 cm)	Normal WC (≤88 cm)	High WC (>88 cm)	Normal WC (≤88 cm)	High WC (>88 cm)
No. of participants	48,862	1,200	33,297	15,782	4,448	34,733
Any cause (n)	11,847	444	7,380	4,898	893	9,507
model 1	1.00(ref)	**1.38(1.26, 1.52)**	**0.91(0.89, 0.94)**	**1.20(1.16, 1.24)**	**0.92(0.86, 0.99)**	**1.29(1.25, 1.32)**
model 2	1.00(ref)	**1.34(1.22, 1.48)**	**0.90(0.87, 0.93)**	**1.16(1.12, 1.20)**	**0.90(0.84, 0.96)**	**1.22(1.19, 1.26)**
model 3	1.00(ref)	**1.26(1.15, 1.39)**	**0.91(0.88, 0.93)**	**1.12(1.08, 1.16)**	**0.93(0.86, 0.99)**	**1.21(1.18, 1.25)**
CVD (n)	2,976	104	2,019	1,389	266	2,785
model 1	1.00(ref)	**1.24(1.02, 1.51)**	1.00(0.94, 1.05)	**1.31(1.23, 1.40)**	1.11(0.98, 1.26)	**1.53(1.46, 1.62)**
model 2	1.00(ref)	1.19(0.98, 1.45)	0.97(0.91, 1.02)	**1.25(1.17, 1.33)**	1.06(0.93, 1.20)	**1.41(1.34, 1.49)**
model 3	1.00(ref)	1.14(0.94, 1.39)	0.97(0.92, 1.03)	**1.21(1.14, 1.29)**	1.08(0.95, 1.22)	**1.40(1.32, 1.48)**
Cancer (n)	3,332	115	281	1,403	258	2,797
model 1	1.00(ref)	**1.35(1.12, 1.63)**	0.96(0.91, 1.01)	**1.26(1.18, 1.34)**	0.90(0.79, 1.02)	**1.26(1.20, 1.33)**
model 2	1.00(ref)	**1.32(1.10, 1.59)**	**0.95(0.90, 0.99)**	**1.23(1.15, 1.31)**	0.89(0.78, 1.01)	**1.21(1.15, 1.27)**
model 3	1.00(ref)	**1.22(1.02, 1.47)**	0.97(0.92, 1.02)	**1.20(1.12, 1.28)**	0.94(0.83, 1.07)	**1.24(1.17, 1.30)**
Other causes (n)	5,539	225	3180	2,106	369	3,925
model 1	1.00(ref)	**1.48(1.30, 1.69)**	**0.85(0.81, 0.88)**	**1.10(1.05, 1.16)**	**0.84(0.76, 0.94)**	**1.18(1.13, 1.23)**
model 2	1.00(ref)	**1.45(1.27, 1.66)**	**0.83(0.80, 0.87)**	**1.08(1.02, 1.13)**	**0.82(0.74, 0.91)**	**1.14(1.09, 1.19)**
model 3	1.00(ref)	**1.36(1.19, 1.56)**	**0.83(0.80, 0.87)**	1.03(0.98, 1.09)	**0.83(0.75, 0.93)**	**1.11(1.06, 1.16)**

Model 1: age at baseline and race/ethnicity.
Model 2: model 1+ education, income, NSES, unopposed estrogen usage status, and estrogen + progesterone usage status.
Model 3: model 2 + smoking status, physical activity, total energy intake, and AHEI-2010 score.
Bold values are statistically significant at $p < 0.05$.

© 2019 Sun Y et al. *JAMA Network Open.*

eTable 4. Baseline BMI and WHR Status in Association With All-Cause and Cause-Specific Mortality Among 156 624 Postmenopausal Women

	Normal weight (BMI 18.5-24.9 kg/m^2)		Overweight (BMI 25.0-29.9 kg/m^2)		Obesity (BMI ≥30 kg/m^2)	
Causes of death	Normal WHR (<0.85)	High WHR (≥0.85)	Normal WHR (<0.85)	High WHR (≥0.85)	Normal WHR (<0.85)	High WHR (≥0.85)
No. of participants	48498	5627	39007	15902	25093	22497
Any cause (n)	11992	2288		5442	6385	7582
model 1	1.00(ref)	**1.43(1.37, 1.49)**	0.98(0.95, 1.01)	**1.29(1.25, 1.33)**	**1.21(1.17, 1.24)**	**1.60(1.56, 1.65)**
model 2	1.00(ref)	**1.40(1.34, 1.46)**	**0.96(0.93, 0.99)**	**1.24(1.20, 1.28)**	**1.15(1.12, 1.19)**	**1.50(1.46, 1.55)**
model 3	1.00(ref)	**1.33(1.27, 1.39)**	**0.96(0.93, 0.98)**	**1.20(1.16, 1.24)**	**1.16(1.12, 1.19)**	**1.47(1.42, 1.51)**
CVD (n)	3163	657	2682	1806	2077	2580
model 1	1.00(ref)	**1.44(1.33, 1.57)**	**1.06(1.01, 1.11)**	**1.47(1.39, 1.56)**	**1.45(1.37, 1.53)**	**1.98(1.88, 2.08)**
model 2	1.00(ref)	**1.40(1.29, 1.53)**	1.02(0.97, 1.08)	**1.38(1.31, 1.47)**	**1.34(1.27, 1.42)**	**1.79(1.70, 1.89)**
model 3	1.00(ref)	**1.33(1.22, 1.45)**	1.01(0.96, 1.07)	**1.33(1.26, 1.42)**	**1.33(1.25, 1.41)**	**1.74(1.65, 1.84)**
Cancer (n)	3289	552	2670	1446	1812	2059
model 1	1.00(ref)	**1.38(1.26, 1.51)**	1.01(0.96, 1.06)	**1.28(1.21, 1.36)**	**1.14(1.08, 1.21)**	**1.46(1.38, 1.54)**
model 2	1.00(ref)	**1.36(1.24, 1.48)**	1.00(0.95, 1.05)	**1.25(1.17, 1.33)**	**1.11(1.04, 1.17)**	**1.39(1.32, 1.47)**
model 3	1.00(ref)	**1.26(1.14, 1.38)**	1.01(0.96, 1.07)	**1.22(1.14, 1.30)**	**1.15(1.08, 1.23)**	**1.40(1.32, 1.48)**
Other causes (n)	5540	1079	4042	2424	2735	3225
model 1	1.00(ref)	**1.45(1.36, 1.55)**	**0.91(0.88, 0.95)**	**1.20(1.14, 1.26)**	**1.12(1.07, 1.17)**	**1.49(1.42, 1.55)**
model 2	1.00(ref)	**1.43(1.34, 1.53)**	**0.90(0.87, 0.94)**	**1.16(1.10, 1.22)**	**1.08(1.03, 1.13)**	**1.41(1.35, 1.48)**
model 3	1.00(ref)	**1.37(1.28, 1.47)**	**0.89(0.86, 0.93)**	**1.11(1.06, 1.17)**	**1.06(1.01, 1.12)**	**1.36(1.29, 1.42)**

Abbreviation: WHR, waist-to-hip ratio.
Model 1: age at baseline and race/ethnicity.
Model 2: model 1+ education, income, OS/CT, NSES, unopposed estrogen usage status, and estrogen + progesterone usage status.
Model 3: model 2 + smoking status, physical activity, total energy intake, and AHEI-2010 score.
Bold values are statistically significant at $p < 0.05$.

© 2019 Sun Y et al. *JAMA Network Open.*
